# Corticostriatal Regulation of Acute Pain

**DOI:** 10.3389/fncel.2017.00146

**Published:** 2017-05-26

**Authors:** Erik Martinez, Harvey H. Lin, Haocheng Zhou, Jahrane Dale, Kevin Liu, Jing Wang

**Affiliations:** ^1^Department of Anesthesiology, Perioperative Care and Pain Medicine, New York University School of MedicineNew York, NY, United States; ^2^Department of Neuroscience and Physiology, New York University School of MedicineNew York, NY, United States

**Keywords:** corticostriatal, prefrontal cortex (PFC), nucleus accumbens, acute pain, aversion

## Abstract

The mechanisms for acute pain regulation in the brain are not well understood. The prefrontal cortex (PFC) provides top-down control of emotional processes, and it projects to the nucleus accumbens (NAc). This corticostriatal projection forms an important regulatory pathway within the brain’s reward system. Recently, this projection has been suggested to control both sensory and affective phenotypes specifically associated with chronic pain. As this projection is also known to play a role in the transition from acute to chronic pain, we hypothesized that this corticostriatal circuit can also exert a modulatory function in the acute pain state. Here, we used optogenetics to specifically target the projection from the PFC to the NAc. We tested sensory pain behaviors with Hargreaves’ test and mechanical allodynia, and aversive pain behaviors with conditioned place preference (CPP) test. We found that the activation of this corticostriatal circuit gave rise to bilateral relief from peripheral nociceptive inputs. Activation of this circuit also provided important control for the aversive response to transient noxious stimulations. Hence, our results support a novel role for corticostriatal circuitry in acute pain regulation.

## Introduction

Acute pain occurs on a daily basis. Sometimes, acute pain can be associated with trauma such as postoperative pain, or infection such as herpetic zoster pain. In these cases, acute pain can become pathological and impair recovery and rehabilitation from the underlying disease; it can even have the potential to progress to persistent or chronic pain (Kehlet et al., 2006). Peripheral and spinal circuits for acute pain have been well investigated. Brain circuits that regulate acute pain, however, are not well understood beyond the classic descending modulatory pathway involving the projection from the periaqueductal gray (PAG) to the rostral ventral medulla (RVM) which in turn projects to the spinal cord (Fields et al., 1983; Morgan et al., 1989, 1991; Ossipov et al., 2014).

The prefrontal cortex (PFC) is a key region in the brain for top-down control of sensory and affective processes (Ressler and Mayberg, 2007; Fuster, 2009; Arnsten et al., 2012), and it emerges as a possible candidate for pain regulation. An area of the PFC in the human brain, the dorsolateral-PFC (DL-PFC), in particular, is involved in emotional regulation and thought to play a protective role against chronic neuropsychiatric conditions such as depression and anxiety (Tucker et al., 1978; Pascual-Leone et al., 1996; Schlaepfer et al., 2003; Bishop et al., 2004; Balconi and Ferrari, 2012). Recent evidence indicates that with chronic pain, the DL-PFC undergoes gray matter loss and altered functional connectivity with other brain regions (Apkarian et al., 2004; Geha et al., 2008; Moayedi et al., 2011; Kucyi et al., 2014). Meanwhile, animal studies have demonstrated that synaptic changes within the PFC occur in chronic pain models (Apkarian et al., 2005; Metz et al., 2009; Ji et al., 2010; Li et al., 2010; Ji and Neugebauer, 2011; Hung et al., 2014). Furthermore, a recent study has shown that activation of the prelimbic-PFC (PL-PFC), the rodent homolog for the DL-PFC, can also inhibit chronic pain (Lee et al., 2015). It is not known, however, whether or how the PFC regulates acute pain conditions.

The PFC has been shown to project to the nucleus accumbens (NAc) in the context of reward-type behaviors (Beckstead and Norgren, 1979; Sesack et al., 1989; Brog et al., 1993; Ishikawa et al., 2008). This projection to the NAc is thought to play a role in restraining reward-seeking phenotypes. Interestingly, the NAc has been shown to play a role in acute as well as chronic pain regulation (Gear et al., 1999; Becerra et al., 2001; Magnusson and Martin, 2002; Becerra and Borsook, 2008; Geha et al., 2008; Gear and Levine, 2009; Baliki et al., 2010; Goffer et al., 2013; Navratilova and Porreca, 2014). This projection from the PFC to NAc plays an important role in the chronic pain state, as the strength of this connection increases in patients who experience chronic low back pain (Baliki et al., 2012). A recent study further demonstrated that the PFC-NAc projection can effectively alter the chronic pain phenotype (Lee et al., 2015). However, several questions remain. First, what is the role of the PFC-NAc projection in acute pain regulation? Second, the PFC is known to provide bilateral projections to the NAc, but does unilateral or bilateral corticostriatal projection mediate pain-regulatory effects? Finally, what is the role of this circuit in the regulation of acute aversive response to transient pain signals?

In the current study, we used optogenetics to investigate the role of the PFC-NAc projection in acute pain regulation. We used four measures to assess pain behaviors: (1) Hargreaves’ test for acute thermal nociception for normal tissues; (2) mechanical allodynia to test hypersensitivity in the case of acute tissue injury; (3) a classic conditioned place preference (CPP) test to assay the aversive component of acute pain in the context of a surgical incision; and (4) a modified CPP test to assay the aversive component of acute pain without any tissue injury. We specifically activated the excitatory neurons from the PFC that projected to the NAc and found that the activation of this neural circuit diminished behavioral responses to ipsilateral as well as contralateral nociceptive inputs. Furthermore, we found that the activation of this corticostriatal circuit also reduced the aversive response to transient noxious stimulations with or without tissue injury. Thus, this corticostriatal circuit provides a key role in acute pain regulation.

## Materials and Methods

### Animals

All procedures in this study were approved by the New York University School of Medicine Institutional Animal Care and Use Committee (IACUC) as consistent with the National Institute of Health (NIH) *Guide for the Care and Use of Laboratory Animals* (publication number 85-23) to ensure minimal animal use and discomfort. Male Sprague-Dawley rats were purchased from Taconic Farms, Albany, NY, USA and kept at Mispro Biotech Services Facility in the Alexandria Center for Life Science, with controlled humidity, room temperature, and 12-h (6:30 AM to 6:30 PM) light-dark cycle. Food and water were available *ad libitum*. Animals arrived to the animal facility at 250 g and were given on average 10–14 days to adjust to the new environment prior to the onset of any experiments.

### Virus Construction and Packaging

Recombinant adeno-associated virus (AAV) vectors were serotyped with AAV1 coat proteins and packaged by the viral vector core at the University of Pennsylvania. Viral titers were 5 × 10^12^ particles/mL for AAV1.CAMKII.ChR2-eYFP.WPRE.hGH and AAV1.CAMKII.eYFP.WPRE.hGH.

### Stereotaxic Cannula Implantation and Intracranial Viral Injections

As described previously (Goffer et al., 2013; Lee et al., 2015), rats were anesthetized with Isoflurane (2%). Virus was delivered to the PL-PFC only. Briefly, rats were bilaterally injected with 0.6 μL of AAV1.CAMKII.ChR2-eYFP.WPRE.hGH or AAV1.CAMKII.eYFP.WPRE.hGH slowly using a 32 gauge 5 μL Hamilton syringe at AP: +2.9 mm; ML: ±1.6 mm; DV: −3.7 mm with tips angled 12.5° toward the midline. The microinjection needles were left in place for an additional 10 min to allow for diffusion of virus particles away from the injection site and to minimize spread of viral particles along the injection tract. Rats were then implanted with 200 μm optic fibers held in 2.5 mm ferrules (Thorlabs) in the PL-PFC: AP +2.9 mm, ML ±1.6 mm, DV −2.7 mm with tips angled 12.5° toward the midline. For the NAc core, rats were stereotaxically implanted with two optic fibers bilaterally: AP +2.2 mm, ML ±2.8 mm, DV −5.7 mm with tips angled 12° toward the midline. Optic fibers were held in place by dental acrylic.

Following animal sacrifice, cryogenic brain sections were collected at a thickness of 20 μm using Microm HM525 Cryostat and analyzed for cannula localization with histological staining. Animals with improper cannula placements, low viral expression, or viral expression outside the PL-PFC were excluded from the study.

### Immunohistochemistry

Rats were deeply anesthetized with Isoflurane and transcardially perfused with ice-cold PBS followed by 4% paraformaldehyde (PFA) in PBS. Brains were fixed in PFA overnight and then transferred to 30% sucrose in PBS to equilibrate for 3 days as described (Lee et al., 2015). Twenty micrometer coronal sections were made with a cryostat and washed with PBS for 10 min. Sections were washed in PBS and coverslipped with Vectashield mounting medium. Sections were also made after viral transfer for opsin verification, and were stained with anti-rabbit GFP (1:500, Abcam, Cambridge, MA, USA, #AB290), NeuN (1:200, Vector Laboratories, Burlingame, CA, USA), DAPI (Vector Laboratories, Burlingame, CA, USA), and CaMKII-α (6G9) mouse mAb (1:100, Cell Signaling Technology, Danvers, MA, USA #50049) antibodies. Secondary antibodies were anti-rabbit IgG conjugated to AlexaFluor 488, and anti-mouse IgG conjugated to AlexaFluor 647 (1:500, Life Technologies, Carlsbad, CA, USA). Images were acquired with a Zeiss LSM 700 Confocal Microscope (Carl Zeiss, Thornwood, NY, USA).

### Paw Incisional (PI) Procedure

The paw incisional (PI) surgery was performed as described previously (Brennan et al., 1996; Su et al., 2015). Rats were anesthetized with Isoflurane anesthesia (2%), and the plantar surface of the right hind paw was sterilized and prepared. A 1.5 cm longitudinal incision was made with a number 10 scalpel through skin and fascia of the right plantar aspect of the paw. The incision started 0.5 cm from the proximal end of the heel, extending to the mid-paw. The plantaris muscle was elevated and incised longitudinally. Pressure was applied to stop bleeding, and the superficial wound was opposed with three single sutures using 5–0 nylon. The animals were allowed to recover in their home cages. Control (sham-treated) rats received Isoflurane anesthesia without the paw incision procedure.

### Animal Behavioral Tests

Animals used for behavior received either AAV1.CAMKII.ChR2-eYFP.WPRE.hGH or AAV1. CAMKII.eYFP.WPRE.hGH (control group) in the PL-PFC. Behavioral tests with optogenetic stimulation in the PFC were done 2 weeks after viral injection. Tests with stimulation in the NAc core were done 4 weeks after injection to ensure optimal expression of opsins.

Prior to behavioral tests, optic fibers were connected to a 473 nm laser diode through an FC/PC adapter (Shanghai Dream Lasers, Shanghai). Laser intensity was measured with a power meter (Thorlabs, Newton, NJ, USA) prior to behavior testing. The laser was delivered using a TTL pulse-generating box (Tucker-Davis Technologies, Alachua, FL, USA). A laser protocol that included alternating light-on and light-off epochs for 30 s each was provided for the duration of the mechanical allodynia test, Hargreaves’ test, and CPP test. Within the light-on epoch, the laser was pulsed at 20 Hz with 10 ms pulse length.

#### Mechanical Allodynia Test

A traditional Dixon up-down method with von Frey filaments of logarithmically incremental stiffness (0.45, 0.75, 1.20, 2.55, 4.40, 6.10, 10.50, 15.10 g) was used to measure mechanical hypersensitivity as described previously (Chaplan et al., 1994; Bourquin et al., 2006; Wang et al., 2011; Su et al., 2016). Rats were individually placed in plexiglass chambers over a mesh table and acclimated for 30 min. Fifty percent withdrawal thresholds were calculated as described previously (Wang et al., 2011). von Frey filaments were applied vertically to the plantar surface of the hind paw, adjacent and medial to the incision as described previously (Brennan et al., 1996; Su et al., 2015).

#### Hargreaves’ Test (Plantar Test)

The Hargreaves’ test was performed to evaluate the response to acute thermal stimulation (Tawfic et al., 2014). We used a mobile radiant heat-emitting device with an aperture of 10 mm in diameter (37370-Plantar Test, Ugo Basile, Italy) to produce acute noxious thermal stimuli and measure the latency to paw withdrawal. Rats were placed individually in a clear plastic chamber and left to acclimate prior to testing. The mobile heat generator was aimed at the plantar surface of the rat’s hind paw, and an infrared intensity of 40 was used. The latency to paw withdrawal was recorded automatically. Paw withdrawals due to locomotion or weight shifting were not counted and the trials were repeated. Measurements were repeated five times at 5 min intervals on the right paw. Separate trials were conducted for baseline (no activation), bilateral, contralateral and ipsilateral optogenetic activation. The averages of the five measurements for each trial were taken and analyzed.

#### Conditioned Place Preference (CPP)

CPP experiments were conducted in a standard three-compartment apparatus (Stoelting co., Wood Dale, IL, USA) consisting of two large compartments of equal size joined by a tunnel (Lee et al., 2015). Rat movements were recorded by a camera and analyzed with ANY-maze software. The CPP protocol was modified from King et al. (2009), and it included preconditioning, conditioning, and testing phases. Preconditioning was performed across 2 days for PI-treated rats. During preconditioning, animals were exposed to the environment with full access to all chambers for 30 min each day. On day 2, the movement of each rat was recorded for 15 min and analyzed to verify the absence of any preconditioning chamber preference. Animals spending more than an 80% (time spent >720 s) or less than 20% (time spent <180 s) of the total time in any chamber were eliminated from further testing or analysis (approximately 15% of total animals), as in previous studies (King et al., 2009; De Felice et al., 2013; Lee et al., 2015). Following the pre-conditioning phase, rats underwent paw incision. Starting 1 day after incision, rats underwent conditioning for two consecutive days with alternating treatment-chamber pairings in the morning and afternoon. During conditioning, rats were placed in the paired chamber without access to the other compartments in the presence or absence of light treatment for 30 min. Half of the rats received light (laser) treatment-chamber pairing in the morning and no light treatment-chamber pairing in the afternoon. The other half of the rats received opposite treatments. Both light vs. no light treatments and chamber pairings were counterbalanced, and at least 4 h separated morning and afternoon sessions. On the test day, the animals were placed into the neutral (conduit) chamber and had access to all chambers for a total of 15 min with no light treatments. During these 15 min, animal movements in each of the chambers were recorded, and the time spent in either of the treatment chambers was analyzed by ANY-maze software. Increased time spent in a chamber associated with either light or no light treatment indicates preference for that chamber.

For acute pain CPP on naïve rats, we modified the classic CPP with a single day protocol (Johansen et al., 2001; Johansen and Fields, 2004; King et al., 2009; De Felice et al., 2013; Lee et al., 2015). This modified CPP protocol included preconditioning (baseline), conditioning and testing phases (10 min during each phase). Conditioning boxes were positioned on top of a metal mesh stand during all three phases (preconditioning, conditioning and testing). The phases occurred one immediately after the other. Animals spending more than 80% or less than 20% of the total time in either chamber in the preconditioning phase were eliminated from further analysis (approximately 20% of total animals). Immediately following the pre-conditioning phase, the rats underwent conditioning for 10 min. In both chambers, rats received a needle prick to a hind paw with a 27 g needle. This noxious mechanical stimulus was repeated every 10 s. Optogenetic activation was paired with one of the treatment chambers, and no light treatment was paired with the opposite chamber. Optogenetic stimulation and chamber pairings were counterbalanced. During the test phase, the animals did not receive any noxious stimulation or optogenetic treatment and had free access to both compartments for a total of 10 min. Animal movements in each of the chambers were recorded, and the time spent in either of the treatment chambers was analyzed by ANY-maze software. A preference score was calculated by subtracting the amount of time a rat stayed in the chamber associated with optogenetic activation during the test phase by the amount of time it stayed in that chamber at baseline.

### Statistics

The results of behavioral experiments were given as mean ± SEM. For mechanical allodynia, a two-way ANOVA with repeated measures and *post hoc* multiple pair-wise comparison Bonferroni tests were used to compare the 50% withdrawal threshold for PI- treated and control rats. A two-way ANOVA was also used to compare the withdrawal threshold in PI- treated and control rats with either channelrhodopsin-2 (ChR2) or enhanced yellow fluorescent protein (eYFP)-only treatment. A two-tail paired Student’s *t* test was used to analyze the results from the Hargreaves’ test. For CPP tests in PI treated rats, differences in time spent in each chamber before conditioning (pre-conditioning) and after conditioning (test) were analyzed using a two-way ANOVA with repeated measures followed by *post hoc* Bonferroni tests. A one-way ANOVA was used to compare differences in preference scores during the acute pain CPP test. For all tests, a *p* value <0.05 was considered statistically significant. All data were analyzed using GraphPad Prism Version 7 software (GraphPad, La Jolla, CA, USA).

## Results

### Activation of the PFC Relieves Acute Pain

**Figure 1:**
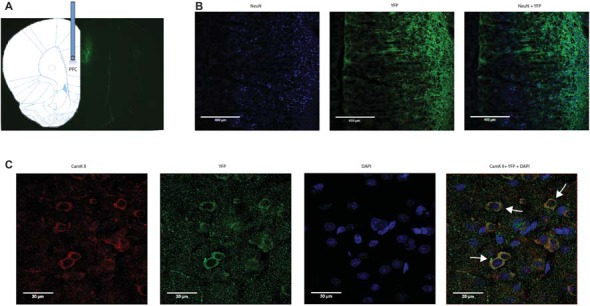
**Functional expression of channelrhodopsin-2 (ChR2) in prefrontal cortex (PFC) neurons. (A)** A representative brain slice shows histologic expression of ChR2-enhanced yellow fluorescent protein (eYFP) in the prelimbic region of the PFC (PL-PFC) 2 weeks after viral injection. ChR2-eYFP expression and fiber location were found to be restricted to neurons in the PL-PFC region. **(B)** Images of PL-PFC at 20x magnification. Neurons were co-stained with NeuN and YFP. **(C)** Images taken of PL-PFC stained with CAMKII, YFP, and DAPI at 100x magnification. Arrows indicate co-staining of CAMKII and YFP.

We applied an optogentic approach to study the role of the PFC in acute pain regulation. We focused on the PL-PFC, which is functionally related to the DL-PFC in humans, a region known for top-down control of emotional processes. We injected AAV encoding light sensitive ChR2 fused to ChR2-eYFP (Fenno et al., 2011; Witten et al., 2011; Nelson et al., 2013; Schneider et al., 2014) into neurons of the PL-PFC (Figure 1). The ChR2 expression was driven by a CAMKII promotor to specifically target pyramidal neurons in the PFC. Two weeks after viral injections, we found stable histologic expression of ChR2-eYFP that is restricted to the PL-PFC (Figure 1A). Co-staining with NeuN, a neuronal marker, and CAMKII, a marker for pyramidal neurons, confirms that ChR2s are predominantly expressed in these excitatory neurons (Figures 1B,C).

**Figure 2:**
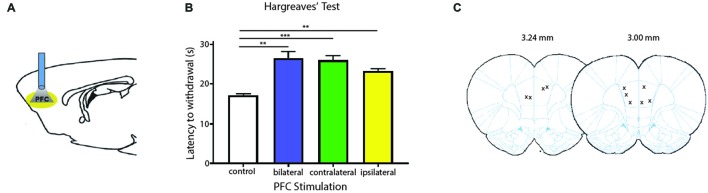
**Activation of PL-PFC neurons relieves acute pain. (A)** Schematic showing the optogenetic strategy to activate PL-PFC neurons. **(B)** Bilateral (*n* = 5, *p* = 0.0064), contralateral (*n* = 5, *p* = 0.0008) and ipsilateral (*n* = 5, *p* = 0.0041) activation of PFC neurons caused an increase in the latency to paw withdrawal during Hargreaves’ test when compared to control (*n* = 5). Two-tailed paired Student’s *t* test. Error bars show mean and SEM. **(C)** Diagram showing the locations of optic fibers in the PL-PFC.

We next tested the role of the PFC in acute pain regulation. We first assessed the influence of the PFC on acute nociception using the Hargreaves’ test (Figure 2). The Hargreaves’ test allowed us to assess withdrawal reflex in response to an acute noxious stimulus, and thus it provided a quantification for acute nociception in the absence of any tissue injury. We found that bilateral activation of the PFC significantly increased the latency to paw withdrawals (Figure 2B). The PFC is known to provide bilateral projection to limbic areas to regulate affect and mood. It is also known to project to the PAG to provide descending inhibitory control on spinal dorsal horn neurons. However, the contribution of PFC activation ipsilateral or contralateral to acute nociceptive inputs has not been well studied. To our surprise, activation of the PFC either ipsilateral or contralateral to the side of noxious stimulation was sufficient to provide similar analgesic effects as bilateral activation. These results argue that at the prefrontal level, in terms of descending pain control, there is functional redundancy with respect to laterality.

**Figure 3:**
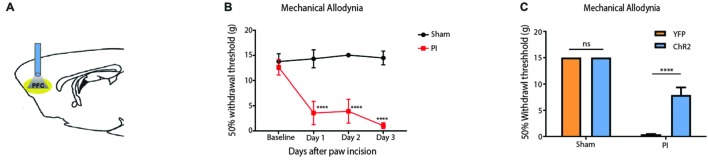
**Activation of the PFC relieves acute incisional pain. (A)** Schematic showing fiber placement and optogenetic activation of PL-PFC neurons during mechanical allodynia tests. **(B)** Paw incision caused mechanical allodynia, compared with sham procedure involving only anesthesia treatment without incision. *n* = 6, *p* < 0.0001. A two-way ANOVA with repeated measures and *post hoc* multiple pair-wise comparison Bonferroni test was used to compare the 50% withdrawal threshold for paw incisional (PI)- treated and control rats. **(C)** Light activation of ChR2-expressing neurons in the PL-PFC decreased mechanical allodynia in PI-, but not sham-treated rats. Light treatment did not have any effect on animals which received vectors containing YFP-only. Two-way ANOVA with Bonferroni post-test, *n* = 7–8, *p* < 0.0001. Light treatment was performed at least 2 weeks after PI/sham procedures. Error bars show mean and SEM.

To validate our results on the Hargreaves’ test, we tested the effect of PFC activation on pain phenotypes associated with an acute pain model—the paw incision (PI) model (Figure 3). We incised the hind paws of rats to mimic acute incisional pain. Mechanical allodynia was used to assess the acute nociceptive response associated with tissue injury, an important measure of acute pain phenotypes (Kehlet et al., 2006; Basbaum et al., 2009). We found that mechanical allodynia lasted 3 days (Figure 3B), compatible with earlier reports (Su et al., 2015). When we shone light to activate the PFC, it significantly reduced mechanical allodynia. Control rats injected with YFP only did not demonstrate such pain relief (Figure 3C). Thus, PFC activation not only inhibits acute withdrawal response to noxious stimulations in normal tissues, it also inhibits acute evoked pain resulting from hypersensitivity at the site of tissue injury.

**Figure 4:**
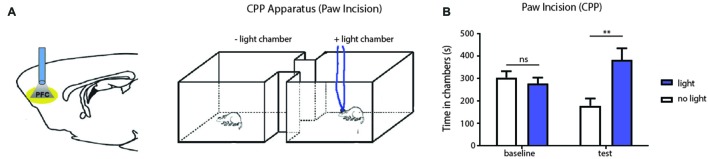
**PFC activation reduces affective symptoms of pain in the PI model. (A)** Schematic showing optogenetic activation of PFC neurons during the conditioned place preference (CPP) for PI-treated rats. One chamber was paired with optogenetic activation, and the other chamber was paired without light activation. **(B)** CPP results showing that PI rats, after conditioning, demonstrated a significant preference for the chamber associated with PFC activation. *n* = 7, *p* < 0.0012. Two-way ANOVA with repeated measures and Bonferroni post-test. Error bars show mean and SEM. **p < 0.01.

The PFC is known to regulate both sensory perception and affective behaviors (Ressler and Mayberg, 2007; Arnsten et al., 2012). We next investigated whether activation of the PFC can also relieve affective symptoms of pain, focusing in particular on the aversive quality of pain. CPP is an established assay to capture the negative reinforcement associated with pain relief and to unmask the aversive quality of ongoing pain (King et al., 2009; Uprety et al., 2014). While the CPP has been most often used in chronic pain settings, we used this test to assess the impact of PFC activation on the PI model, which produces pain symptoms of relatively shorter duration. During the conditioning phase, we applied light stimulation in one chamber, and no light in the opposite chamber (Figure 4A). We conditioned rats on days 1 and 2 after paw incision. After conditioning, we found that PI-treated rats demonstrated a significant preference for the chamber associated with PFC activation (Figure 4B). These data indicate that activation of the PFC also provides relief from the aversive aspect of pain transiently associated with PI. Taken together, our results from Hargreaves’ tests, allodynia and CPP demonstrate the ability of the PFC to relieve acute pain.

### Activation of the Corticostriatal Circuit Inhibits Acute Pain

We next investigated the projection from the PFC to the NAc core in the regulation of acute pain. PL-PFC is known to project strongly to the NAc core (Sesack et al., 1989). A previous study has shown that this projection can relieve sensory allodynia and aversive learning associated with chronic neuropathic pain (Lee et al., 2015). Chronic neuropathic pain, however, is known to alter synaptic circuits within the PFC and NAc (Li et al., 2010; Goffer et al., 2013; Koga et al., 2015; Su et al., 2015). Thus, it is possible that the analgesic effect of corticostriatal activation may be specific or restricted to chronic pain conditions. We set out to investigate if this circuit can be activated to reduce symptoms of acute pain as well.

**Figure 5:**
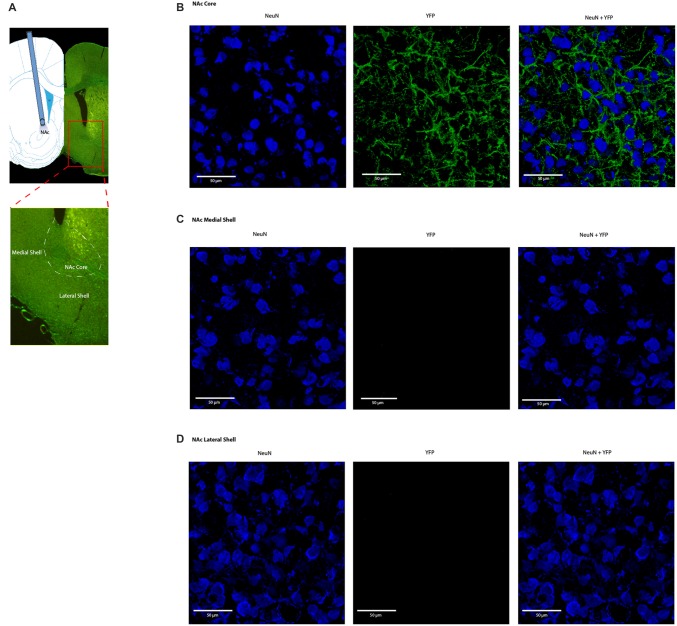
**Expression of ChR2-eYFP in the axon terminals of prefrontal neurons that project to the nucleus accumbens (NAc) core. (A)** Representative brain slice showing ChR2-eYFP expression in the NAc core 4 weeks after PL-PFC injection. **(B)** Higher magnification views demonstrating expression of ChR2 in prefrontal neurons that project to the NAc core. **(C,D)** Higher magnification views demonstrating a lack of opsin expression in the NAc medial and lateral shell.

**Figure 6:**
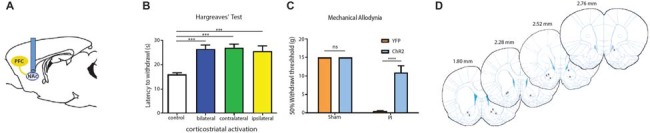
**PFC neurons project to the NAc core to relieve acute pain. (A)** Schematic showing fiber placement and optogenetic activation of NAc core neurons. ChR2 was injected into the PL-PFC. Four weeks later, we activated the PFC-NAc projection using fibers implanted in the NAc core. **(B)** Bilateral (*n* = 7, *p* < 0.0007), contralateral (*n* = 7, *p* < 0.001) and ipsilateral (*n* = 7, *p* = 0.0009) activation of prefrontal neurons that project to the NAc core caused an increase in the latency to paw withdrawal during Hargreaves’ test when compared to control (*n* = 7). Two-tailed paired Student’s *t* test. **(C)** Light activation of the PFC-NAc circuit decreased mechanical allodynia in PI-, but not sham-treated rats. Light treatment did not have any effect on animals which received vectors containing YFP-only *n* = 4–10, *p* < 0.0001. Two-way ANOVA with Bonferroni post-test. Light activation was performed 4 weeks after PI/sham procedures. Error bars show mean and SEM. **(D)** Diagram showing the locations of optic fibers in the NAc core. ***p < 0.001, ****p < 0.0001.

Four weeks after we injected ChR2-eYFP into the PL-PFC, we found its expression in the axon terminals of prefrontal neurons that project to the NAc core (Figures 5A,B). In contrast, we did not observe significant expression in the NAc shell, demonstrating that the PL-PFC projects primarily to the NAc core (Figures 5C,D). These results are in agreement with previous findings (Beckstead and Norgren, 1979; Sesack et al., 1989; Sesack and Pickel, 1992; Brog et al., 1993; Vertes, 2004). We photoactivated the ChR2 which was expressed at the prefrontal axon terminals using optic fibers inserted in the NAc core (Figures 6A,D). This approach allowed us to specifically target the PL-NAc core projection. We first tested the effect of this corticostriatal activation on acute thermal nociceptive regulation using Hargreaves’ tests. We found that optogenetic stimulation resulted in a significant increase in the latency to paw withdrawal (Figure 6B). The PFC provides bilateral projections to the NAc, and here we found that ipsilateral or contralateral activation of the PFC-NAc projection provided pain relief to the same extent as bilateral stimulation. These results demonstrate, for the first time, that output from NAc of either side is sufficient to confer analgesic effects.

We then tested if the activation of this projection can also relieve sensory hypersensitivity associated with acute post-incisional pain. Using the PI model, we found that photoactivation of prefrontal neurons that project to the NAc resulted in significant anti-allodynic effects, to the same level as direct activation of the PFC (Figure 6C). In contrast, as expected, light treatment did not have any noticeable effects on sham-treated (control) rats.

**Figure 7:**
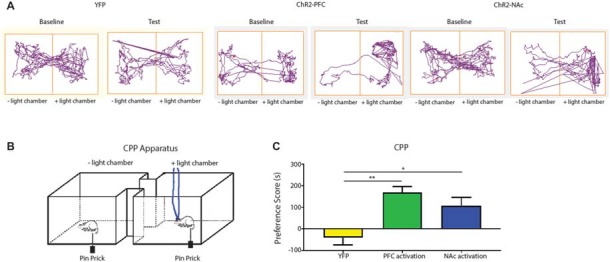
**Activation of the corticostriatal circuit regulates the aversive response to acute pain. (A,B)** Experimental set up for CPP test for acute pain. In each chamber, rats received peripheral noxious stimulation. One of the chambers was paired with optogenetic treatment; the other chamber was paired with no treatment. Rat movement was recorded with ANY-maze software. **(C)** A comparison of preference scores from control activation (using YFP only without opsin expression), activation with ChR2 in the PFC, and activation with ChR2 in the NAc. *n* = 6, *p* = 0.0016 comparing YFP with PFC activation; *p* = 0.021 comparing YFP with NAc activation. One-way ANOVA with Bonferroni post-test. Error bars show mean and SEM. *p < 0.05, **p < 0.01.

Aversion is a key component of the behavior response to pain. Previous studies have relied on persistent pain models to elicit the aversive response to pain or the negative reinforcement of analgesics (Johansen et al., 2001; Qu et al., 2011; Barthas et al., 2015; Navratilova et al., 2015). We are interested, however, in assessing the aversive response to transient noxious stimulations and the effect of the corticostriatal pathway on the regulation of such response. In order to fully assess the aversive response specific to acute pain in the absence of persistent pain or tissue injury, we modified the traditional CPP assay with a 1-day protocol using a two chamber device. We established baseline preference for 10 min. For all three phases (pre-conditioning, conditioning, and testing), we placed the chambers over a mesh table. During the conditioning phase, we used needle pricks to the right hind paw to produce acute mechanical pain in both chambers; we repeated this stimulus every 10 s for a total of 10 min. We then paired one chamber with photoactivation of either the PFC or the PFC-NAc projection, and the other chamber with no light treatment (Figures 7A,B). Rats were allowed to freely move between the chambers. During the test phase, we removed both peripheral noxious stimulation and optogenetic activation and recorded the amount of time rats spent in either chamber. An increase in the time spent in a particular chamber indicated preference for the treatment associated with that chamber. We calculated a preference score by subtracting the time rats spent in the chamber paired with light treatment at baseline from the time they spent in that chamber during the test phase (Johansen et al., 2001; Johansen and Fields, 2004; De Felice et al., 2013). This preference score has been used in previous studies to provide a quantitative measure for the aversive component associated with persistent pain or the effect of analgesics on relieving the aversive component of chronic pain (Johansen et al., 2001; Johansen and Fields, 2004; De Felice et al., 2013). Here we used this preference score to indicate the ability for activation of the corticostriatal projection to provide relief of the aversive quality of acute pain. We compared the effect of PFC activation with the effect of activation of the PFC-NAc projection on the aversive respose to pain. We found that light activation of the PFC was able to provide a significant amount of reduction in pain aversion, as indicated by the preference score, when compared with YFP activation (laser activation of neurons expressing YFP but not ChR2). Furthemore, activation of the corticostriatal circuit provided a similar degree of relief in pain aversion as direct activation of the PFC (Figure 7C). These results suggest that the projection from the PFC to the NAc can provide key regulation for the aversive experience during an acute pain episode.

## Discussion

The PFC is known to provide top-down control for a number of affective processes. Its projections to the PAG, thalamus and amygdala have been studied in the context of chronic pain (Bushnell et al., 2013; Cardoso-Cruz et al., 2013; Ji and Neugebauer, 2014). There is recent evidence that connections between the PFC and the NAc—an important projection within the reward circuitry (Koob and Volkow, 2010)—are distinctly altered by chronic pain (Baliki et al., 2012). In this study, we have shown that this corticostriatal circuit has the capacity to powerfully impact both sensory and affective aspects of the acute pain experience.

Previous studies have used electrical stimulation to demonstrate the anti-nociceptive effects of prefrontal activation (Hardy, 1985; Hardy and Haigler, 1985). Using optogenetics, we are able to target pyramidal neurons in the PFC specifically. Furthermore, we are able to target distinct prefrontal neurons that project to the NAc core. The NAc has been suggested to project both to the PAG and to the brain stem (Yu and Han, 1990; Gear et al., 1999; Becerra et al., 2001; Magnusson and Martin, 2002; Becerra and Borsook, 2008; Geha et al., 2008; Gear and Levine, 2009; Baliki et al., 2010; Goffer et al., 2013). Based on the understanding of the descending projections from the NAc, we speculate that the projection from the PFC to the NAc may potentially trigger downstream pathways via the PAG or brain stem to achieve descending pain inhibition during acute nociceptive episodes. It would be important to test such speculations in future studies.

A second important role of the corticostriatal projection in our study is its regulation of pain aversion. Aversive responses to pain can be assessed by the CPP test (Johansen et al., 2001; Johansen and Fields, 2004; King et al., 2009; De Felice et al., 2013). Previous studies on CPP have relied on spontaneous nociceptive inputs at the site of chronic pain (often induced by tissue or nerve injury) as conditioning signals. Hence, results from these studies are best interpreted in the context of persistent pain. Few studies have examined the temporary aversive evaluation of acute pain signals in the absence of a persistent pain or tissue injury. Our assay here, utilized transient mechanical nociceptive stimuli as a conditioning signal to allow us to assess the circuit mechanisms that regulate such acute aversive evaluation. This design allowed us to demonstrate that the projection from the PFC to the NAc can regulate the aversive response to pain both in the presence of tissue injury resulting from paw incision and, equally importantly, to acute noxious stimulations in the absence of any persistent pain or injury. Therefore, these results strongly suggest that the corticostriatal circuit plays a key role during the experience of acute pain episodes.

The NAc projects to the ventral pallidum and substantia nigra (Nestler and Carlezon, 2006). These projections terminate in the ventral anterior, dorsal and lateral thalamus, which in turn project to parts of the PFC and anterior cingulate cortex (ACC), forming a striato-thalamo-cortical loop. Extensive studies have shown that this loop can regulate reward-based learning (Russo and Nestler, 2013). Our results here demonstrate that the NAc can also regulate the aversive response to pain. Future studies are needed to investigate the outputs from the NAc in the regulation of aversion-based learning in the context of pain, including the possible involvement of this striato-thalamo-cortical loop.

Another important aspect of our study concerns the laterality of the PFC-NAc projection in pain regulation. Whereas, the lateral spinothalamic tract is known to transmit contralateral nociceptive signals from the periphery to the somatosensory cortex, medial pain pathways can carry bilateral nociceptive signals to the medial thalamus which in turn projects to the prefrontal areas. In addition, the PFC can receive bilateral nociceptive information from the somatosensory cortex. Thus, nociceptive inputs to the PFC can be bilateral. The laterality of the descending inhibitory outputs from the PFC is less well documented. In the context of rewards, the PFC projects bilaterally to the NAc. Our results here suggest that the PFC also provides bilateral outputs to the NAc to regulate acute pain. Optogenetic activation of the PFC either ipsilateral or contralateral to the site of nociceptive inputs provides the same degree of pain relief as bilateral activation. Hence, our results indicate that in fact there may be functional redundancy at the level of the cortex. Downstream from the PFC, the NAc can also be activated optogenetically to provide bilateral, ipsilateral and contralateral pain relief. Interestingly, a number of human imaging studies have shown that chronic pain can cause bilateral gray matter atrophy in the PFC as well as the NAc (Baliki et al., 2008, 2012; Rodriguez-Raecke et al., 2009; Fritz et al., 2016). On the other hand, the initial phase in the development of chronic pain has been associated with increased bilateral connectivity of the PFC-NAc circuit (Baliki et al., 2012). Putting our results in the context of these human studies, the corticostriatal circuit may be an important mechanism to regulate acute pain signals. Thus, initially as acute pain transitions to chronic pain, there is strengthening of the bilateral connections between the PFC and the NAc in order to regulate pain. As pain progresses further into the chronic phase, however, through a mechanism not yet known, there appears to be structural and function deficits within the PFC and NAc, leading to a deficiency in the ability of this pathway to regulate pain. This deficit in pain regulation may then contribute to chronic pain phenotypes. In the future, it will be important to probe the mechanisms for such structural and function deficits within this important pain-regulatory circuit.

It should be noted that the PFC is an anatomically and functionally heterogenous region. In rodents, it can be largely defined by the ACC, PL and infralimbic (IL) componenents. The results here demonstrate that activation of the PL region of the PFC can relieve the aversive component of pain, and it can also reduce the sensory evoked pain behaviors such as paw withdrawals. Our results are in agreement with previous results that showed stimulation of the PL-PFC with electrical, pharmacological and optogenetic methods can influence evoked pain behaviors (Hardy, 1985; Hardy and Haigler, 1985; Lee et al., 2015; Wang et al., 2015; Zhang et al., 2015). Other studies have shown that stimulation of the IL region has similar pain-relieving properties (Kiritoshi et al., 2016). However, studies in the ACC and adjacent areas have demonstrated complex and sometimes inconsistent effects of stimulation on evoked pain behaviors (Bissiere et al., 2008; Li et al., 2010; Barthas et al., 2015; Kang et al., 2015). Furthermore, studies have shown that pharmacological stimulation of the PFC (including the ACC and some overlapping PL region) produced aversive learning on its own (Johansen and Fields, 2004). Thus, the anatomic divisions of the PFC likely give rise to a diverse set of functions in the context of pain. In addition, it is possible that different stimulation protocols may play a role in the functional outcomes. Whereas pharmacological modifications of the PFC can produce stable effects that last for hours, optogenetic manipulations tend to result in brief and reversible outcomes. Finally, PL-PFC, the focus of the current study, is known to project specifically to the core subregion of the NAc, whereas IL-PFC projects only weakly to the NAc shell (Beckstead and Norgren, 1979; Sesack et al., 1989; Sesack and Pickel, 1992; Brog et al., 1993; Vertes, 2004). Thus, the circuit projections of various PFC components may play an important role in the functional diversity of this region, and future studies of these projections will inevitably shed further light on the precise regulatory roles for PFC in acute and chronic pain states.

In conclusion, we have shown that the projection from the PL-PFC to the NAc can provide important regulation for the sensory and aversive components of acute pain. Thus, this corticostriatal circuit may be an important therapeutic target for acute pain conditions.

## Author Contributions

EM and HHL contributed equally to this work. EM, HHL, HZ and KL performed behavior testing. JD and HZ performed intracranial surgeries. EM and HZ performed immunohistological staining. EM, HHL and JW performed data analysis. EM assisted in the drafting of the manuscript. JW designed the experiments, supervised the project and drafted the manuscript.

## Conflict of Interest Statement

The authors declare that the research was conducted in the absence of any commercial or financial relationships that could be construed as a potential conflict of interest.
